# Candidate Gene Association Study in Type 2 Diabetes Indicates a Role for Genes Involved in β-Cell Function as Well as Insulin Action

**DOI:** 10.1371/journal.pbio.0000020

**Published:** 2003-10-13

**Authors:** Inês Barroso, Jian'an Luan, Rita P. S Middelberg, Anne-Helen Harding, Paul W Franks, Rupert W Jakes, David Clayton, Alan J Schafer, Stephen O'Rahilly, Nicholas J Wareham

**Affiliations:** **1**Incyte, Palo AltoCaliforniaUnited States of America; **2**Department of Public Health and Primary Care, University of Cambridge Institute of Public HealthCambridgeUnited Kingdom; **3**Diabetes and Inflammation Laboratory, Cambridge Institute for Medical ResearchCambridgeUnited Kingdom; **4**Department of Clinical Biochemistry, University of CambridgeCambridgeUnited Kingdom

## Abstract

Type 2 diabetes is an increasingly common, serious metabolic disorder with a substantial inherited component. It is characterised by defects in both insulin secretion and action. Progress in identification of specific genetic variants predisposing to the disease has been limited. To complement ongoing positional cloning efforts, we have undertaken a large-scale candidate gene association study. We examined 152 SNPs in 71 candidate genes for association with diabetes status and related phenotypes in 2,134 Caucasians in a case-control study and an independent quantitative trait (QT) cohort in the United Kingdom. Polymorphisms in five of 15 genes (33%) encoding molecules known to primarily influence pancreatic β-cell function—*ABCC8* (sulphonylurea receptor), *KCNJ11* (KIR6.2), *SLC2A2* (GLUT2), *HNF4A* (HNF4α), and *INS* (insulin)—significantly altered disease risk, and in three genes, the risk allele, haplotype, or both had a biologically consistent effect on a relevant physiological trait in the QT study. We examined 35 genes predicted to have their major influence on insulin action, and three (9%)—*INSR*, *PIK3R1*, and *SOS1*—showed significant associations with diabetes. These results confirm the genetic complexity of Type 2 diabetes and provide evidence that common variants in genes influencing pancreatic β-cell function may make a significant contribution to the inherited component of this disease. This study additionally demonstrates that the systematic examination of panels of biological candidate genes in large, well-characterised populations can be an effective complement to positional cloning approaches. The absence of large single-gene effects and the detection of multiple small effects accentuate the need for the study of larger populations in order to reliably identify the size of effect we now expect for complex diseases.

## Abstract 2

The absence of large single gene effects and the detection of multiple small effects confirms the genetic complexity of type 2 diabetes and the need for even larger studies

## Introduction

Type 2 diabetes is a serious metabolic disease associated with an increased risk of premature death and substantial disability, largely mediated through its adverse effects on the vasculature. The prevalence of the disease is increasing, and the World Health Organisation estimates suggest that by 2025 there will be 300 million affected individuals worldwide (King et al. 1998). The disorder is characterised by a combination of impaired insulin secretion and insulin action, both of which precede and predict the onset of disease (Weyer et al. 1999). Through its adverse impact on insulin action, obesity is a major risk factor for the disease. Although environmental factors, both post- and prenatal, play an important role in determining the risk of disease, a substantial body of evidence supports the notion that disease susceptibility is influenced by inherited factors (Zimmet 1982). While the molecular basis for several uncommon Mendelian forms of Type 2 diabetes have been defined (Vionnet et al. 1992; Yamagata et al. 1996a, 1996b; Horikawa et al. 1997; Stoffers et al. 1997; Barroso et al. 1999; Malecki et al. 1999; Savage et al. 2002), the nature and range of allelic variants conferring susceptibility to the more common forms of this disorder remain poorly defined. Many investigators have embarked on attempts to identify diabetes susceptibility genes through genome-wide linkage-based approaches using multigenerational pedigrees and/or large numbers of affected sibpairs. Regions of significant linkage, some of which have been replicated in more than one study, have been identified. To date, however, only Calpain 10 (*CAPN10*; LocusLink ID 11132) has emerged from such studies as a new putative diabetogene (Horikawa et al. 2000). While some subsequent studies have supported a role for the *CAPN10* alleles originally described as susceptibility alleles, others have found associations with different alleles and some have found no association with this gene (Baier et al. 2000; Cox 2001; Evans et al. 2001; Hegele et al. 2001; Tsai et al. 2001; Daimon et al. 2002; Elbein et al. 2002; Garant et al. 2002).

The positional cloning effort has been supplemented by a large number of studies examining specific candidate genes using a variety of methodologies, mostly of the case-control association design. Although many positive reports have emerged, few have been consistently replicated. Of these candidates, the most compelling evidence to date, generated from a meta-analysis of multiple published studies, is that a common amino acid variant in the N-terminus of the nuclear receptor peroxisome proliferator-activated receptor γ (*PPARG*; LocusLink ID 5468) confers significant protection against the development of Type 2 diabetes (Altshuler et al. 2000). More recently, evidence has accumulated supporting a role for the E23K variant of *KCNJ11* (LocusLink ID 3767) in Type 2 diabetes predisposition (Hani et al. 1998; Gloyn et al. 2001, 2003; Love-Gregory et al. 2003; Nielsen et al. 2003).

Whole-genome association studies in large case-control populations may ultimately have the greatest power to detect alleles of small but significant effects on the susceptibility to common diseases such as Type 2 diabetes. As yet, however, the resource implications of such an approach are prohibitive. In the meantime, knowledge of both mammalian biology and disease pathogenesis is progressing rapidly, and it is possible to identify a large panel of known genes, the dysfunction of which might reasonably be considered likely to contribute to Type 2 diabetes. In this study we have identified 152 informative single nucleotide polymorphisms (SNPs) in 71 such genes and, using these, have examined their association with Type 2 diabetes and related intermediate phenotypes in Caucasian subjects from the United Kingdom.

## Results/Discussion

### Overall Study Design

Candidate gene studies are based on selection of genes with a known or inferred biological function whose role makes it plausible that they may predispose to disease or the observed phenotype. These types of studies are similar to traditional epidemiological approaches in which an a priori hypothesis between exposure to a given factor (in this case, a genotype at a given locus) and disease is formulated. To date, most Type 2 diabetes candidate gene studies have largely lacked thoroughness and sensitivity, as they have tested a limited number of genes and variants in small populations or in populations that were poorly matched or phenotyped, frequently resulting in a lack of replication of the weak associations detected (Altshuler et al. 2000). Our strategy aimed to address these problems by a unique combination of features, including comprehensive SNP discovery in a large number of candidate genes, testing of a large number of SNPs, use of two independent populations, and analysis of haplotypes in addition to individual SNPs where possible.

**Figure 1:**
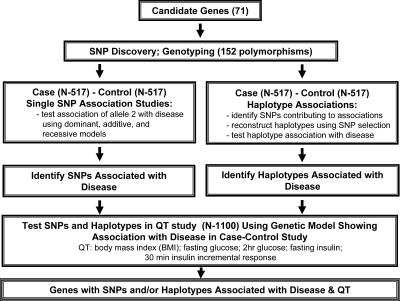
Study Design Candidate genes were selected based on known or putative function. A de novo polymorphism discovery step was undertaken to identify novel variants for association studies. We selected 152 SNPs and tested them in a case-control study and a QT study. Association analysis with Type 2 diabetes was done for SNPs and haplotypes under multiple genetic models. Only SNPs and haplotypes associated with disease were evaluated for association with five diabetes-related QTs under the same model in the QT study.

Figure 1 illustrates the overall design of the study. On the basis of their known or putative role in glucose metabolism, 71 candidate genes were selected for study (Table 1). These were subdivided into three broad groups: (1) genes primarily involved in pancreatic β-cell function; (2) genes primarily influencing insulin action and glucose metabolism in the main target tissues, muscle, liver, and fat; and (3) other genes. This group includes genes that influence processes potentially relevant to diabetes, such as energy intake, energy expenditure, and lipid metabolism. A de novo search for common SNPs was undertaken using fluorescent single-stranded conformation polymorphism (fSSCP) examination of all coding regions and splice junctions in a variety of human populations. All genes were minimally screened against 47 samples of mixed ethnicity, providing 0.99 probability of detecting variants with a minor allele frequency of 0.05. Our ‘in-house' polymorphism detection programme identified 954 SNPs in the 71 genes, with a range of allele frequencies from 0.003 to 0.50. Of the 152 SNPs chosen for further study (Table S1), the great majority had a minor allele frequency of greater than 5%, but in a few instances less frequent variants were typed when the candidate gene had strong biological plausibility and there were no known polymorphisms of higher frequency at the time of SNP selection.

**Table 1:**
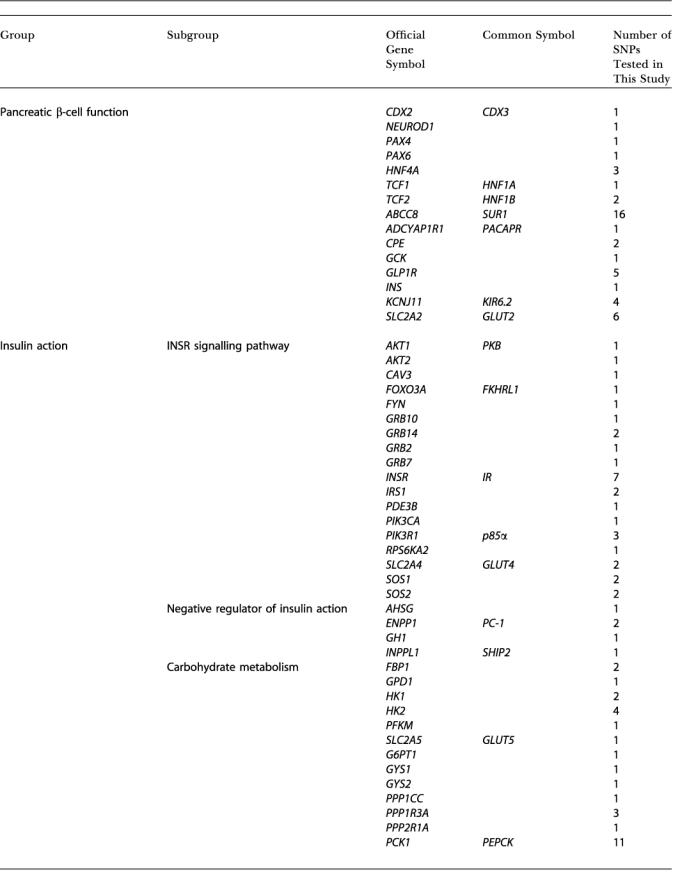
Genes with SNPs Genotyped in This Study

Candidate genes, identified by official HUGO Gene Nomenclature Committee symbols, are grouped by known or putative biological function, with the number of genotyped polymorphisms per gene shown

**Table 1:**
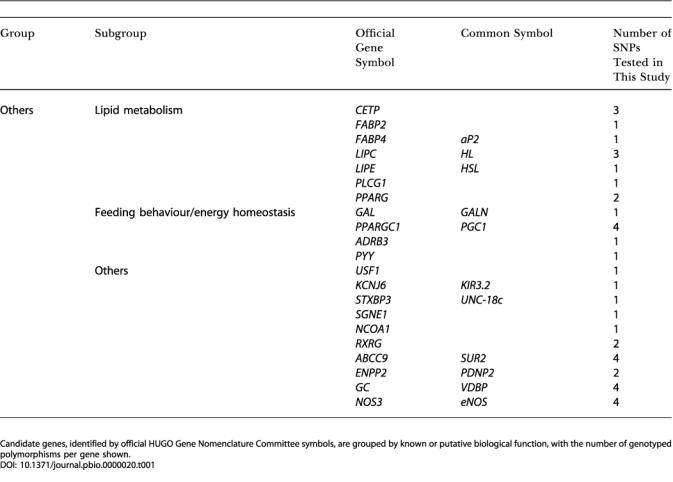
Continued

The 152 SNPs were genotyped in a population-based cohort of 517 unrelated Caucasians in the United Kingdom with Type 2 diabetes and an equal number of controls with normal glycated haemoglobin (HbA1c) levels, individually matched to cases by age, sex, and geographical location. A second independent population was also genotyped for the same 152 SNPs. This consisted of 1,100 middle-aged Caucasian subjects in the United Kingdom who had been extensively and serially phenotyped for glucose tolerance and variables related to insulin secretion, insulin action, and adiposity. In the first stage of data analysis, all SNPs (and haplotypes when multiple SNPs were present at the same gene) were examined for their association with diabetes in the case-control study using multiple models of inheritance. In the second phase of analysis, all SNPs and haplotypes showing statistically significant association with diabetes status in the first phase were examined for association with glucose levels and other intermediate phenotypes in the quantitative trait (QT) study population. The intermediate phenotypes chosen for study were fasting and 2-h post-glucose load plasma glucose levels (measures of glucose tolerance), fasting insulin (a measure of insulin sensitivity), 30-min insulin incremental response (a measure of β-cell function), and body mass index (BMI) (a measure of adiposity). **

**Figure 2:**
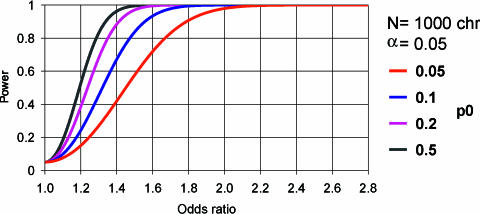
Power Calculations Power of the current Cambridgeshire Case-Control Study to detect associations with risk allele of varying frequencies and with a Type 1 error rate of 5%. Abbreviations: p0, frequency of the predisposing allele; chr, number of chromosomes. Graphs were plotted with the PS power and sample-size program (available at http://www.mc.vanderbilt.edu/prevmed/ps; DuPont and Plummer 1997).

Power to detect an association is dependent on several factors: the frequency of the ‘predisposing' allele, genotype, or haplotype; the accepted false-positive or Type 1 error rate (α); and the odds ratio (OR) or effect size. Rarer alleles, genotypes, or haplotypes with small effects require larger sample sizes to attain the same power to detect an association, as compared to more frequent alleles or alleles with larger effects. At the time that we collected the populations and designed this study, our power calculations had shown that a sample size of 500 cases and 500 matched controls would have 80% power to detected effect sizes as small as 1.3–1.7 OR, depending on the frequency of the predisposing allele, with a 5% Type 1 error rate (Figure 2).

### Overview of Results of Association Studies

**Figure 3:**
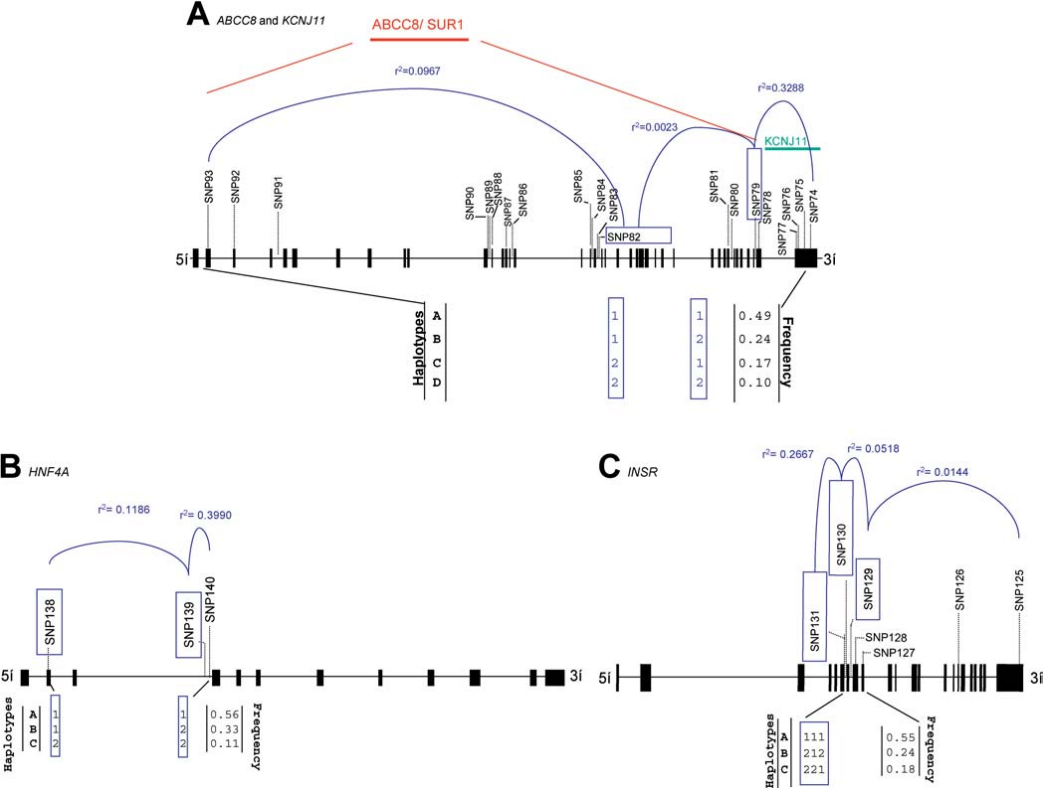
Genes with Haplotypes Associated with Type 2 Diabetes Genomic organization with exons (black boxes or vertical lines) and with genotyped SNPs and SNPs utilised in the haplotype reconstructions (in blue boxes) is shown. The most common haplotypes with population prevalence greater than 5% in the control population are shown, and the measure of LD (*r^2^*) is shown for a subset of the SNPs. (A) *ABCC8–KCNJ11*. (B) *HNF4A*. (C) *INSR*.

Table S1 shows the genotype counts for all 152 SNPs in the case-control and QT studies. In the control subjects, 16 SNPs (10.6%) had a minor allele frequency below 5%; 19 (12.5%) had a minor allele frequency between 5% and 10%; and 117 (76.9%) had a minor allele frequency greater than or equal to 10%. Each variant was tested for association with disease status under several genetic models**. **Twenty SNPs in 11 different genes showed statistically significant association with disease status (*p* < 0.05) under at least one model (Table 2). The strongest statistical evidence for disease association was for genes *SOS1* (LocusLink ID 6654), *SLC2A2* (LocusLink ID 6514), *PIK3R1 (*LocusLink ID 5295), *ABCC8* and *KCNJ11* (LocusLink ID 6833), and *INSR* (LocusLink ID 3643).** Of the 29 loci with multiple SNPs, only three—*HNF4A* (LocusLink ID 3172) *INSR*, and *ABCC8*–*KCNJ11*—showed significant association of particular haplotypes with disease status (Figure 3; Table 3). In only one case (*HNF4A*) was a haplotype significantly associated with disease risk (see below) when no significant association was seen with any individual SNP in that gene. Table S2, shows the results of association studies undertaken in the QT population, further examining the SNPs that had shown significant association in the case-control study. Table 3 shows the relationship between disease-associated haplotypes at *ABCC8*–*KCNJ11*, *HNF4A*, and *INSR* with intermediate phenotypes in the QT study. We now consider in more detail, the data for those genes where the strongest and most consistent effects were seen.

**Table 2:**
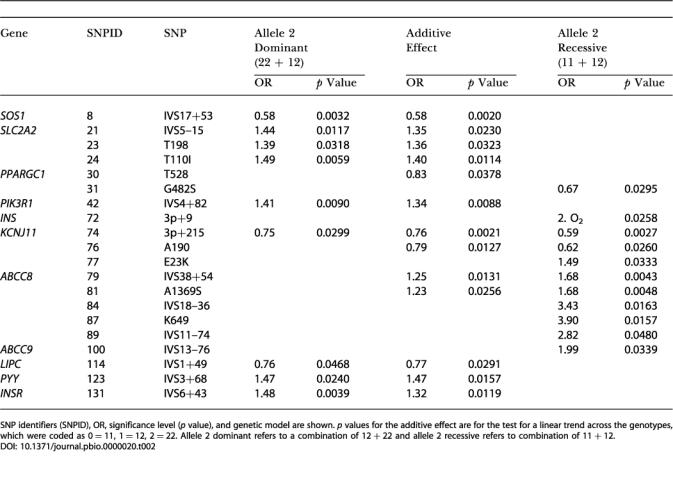
Genes with Variants Significantly Associated with Type 2 Diabetes Status

SNP identifiers (SNPID), OR, significance level (*p* value), and genetic model are shown. *p* values for the additive effect are for the test for a linear trend across the genotypes, which were coded as 0 = 11, 1 = 12, 2 = 22. Allele 2 dominant refers to a combination of 12 + 22 and allele 2 recessive refers to combination of 11 + 12

**Table 3:**
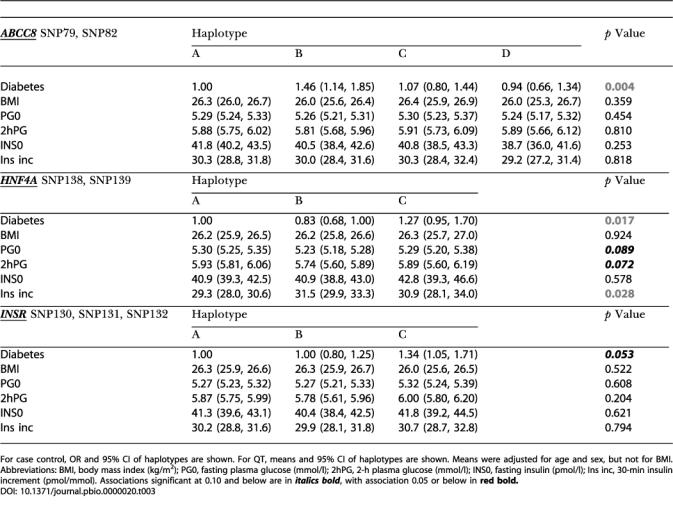
Association of *ABCC8–KCNJ11*, *HNF4A*,** and *INSR* Haplotypes with Diabetes and QTs****

For case control, OR and 95% CI of haplotypes are shown. For QT, means and 95% CI of haplotypes are shown. Means were adjusted for age and sex, but not for BMI. Abbreviations: BMI, body mass index (kg/m^2^); PG0, fasting plasma glucose (mmol/l); 2hPG, 2-h plasma glucose (mmol/l); INS0, fasting insulin (pmol/l); Ins inc, 30-min insulin increment (pmol/mmol). Associations significant at 0.10 and below are in***italics bold***, with association 0.05****** or below in****** **red bold** ***.***

### Genes Primarily Affecting β-Cell Function 

#### *ABCC8* and *KCNJ11* (encoding, respectively, the sulphonylurea receptor and inwardly rectifying potassium channel KIR 6.2).

The genes encoding the two molecular components of the voltage-gated potassium channel of the pancreatic β-cell are located within 4.5 kb of each other on Chromosome 11. *KCNJ11* encodes the channel protein KIR6.2 and *ABCC8* encodes an ATP-binding cassette (ABC) transporter-containing transmembrane protein (SUR1) that is thought to regulate the activity of the channel and that also contains the site to which sulphonylurea antidiabetic drugs bind. Three SNPs in *KCNJ11* were associated with disease under multiple genetic models. The strongest statistical association in this gene was with a 3′-SNP (SNP74; OR 0.59, *p* = 0.0027 under recessive model for allele 2) (see Table 2). In *ABCC8*, five SNPs were associated with disease status under multiple models; the strongest evidence for association with disease were with SNP79 and SNP81, respectively an intronic variant (OR 1.68, *p* = 0.0043; see Table 2) and a missense variant A1369S (OR 1.68, *p* = 0.0048; see Table 2). Although neither of these two SNPs was significantly associated with any trait in the QT study, two other SNPs showed effects in the QT study (see Table S2). SNP84 (IVS18–36) from *ABCC8*, which associated with increased disease risk (OR 3.43, *p* = 0.0163; see Table 2) also associated with increased BMI (mean 28.2 kg/m^2^, 95% confidence interval [CI] [26.6, 29.9] for homozygous 22 versus 26.2 kg/m^2^, 95% CI [25.9, 26.5] for homozygous 11 and 26.3 kg/m^2^, 95% CI [25.7, 26.8] for heterozygous subjects; *p* = 0.016) and associated with borderline significance with higher fasting glucose (5.53 mmol/l, 95% CI [5.29, 5.77] for homozygous 22 versus 5.30 mmol/l, 95% CI [5.26, 5.35] for homozygous 11 and 5.27 mmol/l, 95% CI [5. 19, 5.35] for heterozygous subjects; *p* = 0.057) under a recessive model for allele 2 (see Table S2). SNP87 (K649), which was also significantly associated with increased disease risk (OR 3.90, *p* = 0.0157; see Table 2), also showed borderline significant association with decreased insulin secretion (23.6 pmol/mmol, 95% CI [18.6, 30.1] for homozygous 22 versus 29.8 pmol/mmol, 95% CI [28.4, 31.1] for homozygous 11 and 30.9 pmol/mmol, 95% CI [28.6, 33.4] for heterozygous subjects; *p* = 0.054; see Table S2), consistent with a role for this gene in insulin secretion. Given the close physical proximity of *ABCC8* and *KCNJ11* and their role in the same functional unit, we performed haplotype reconstructions with data from both genes combined (see Figure 3A). Haplotype B was associated with increased disease risk (OR 1.46, 95% CI [1.14, 1.85]; data not shown), but did not show any significant association in the QT study (see Table 3).

Mutations in each of these genes have been associated with familial persistent hyperinsulinaemia hypoglycaemia of infancy (PHHI), a rare disorder of glucose homeostasis characterised by up-regulated insulin secretion despite severe hypoglycaemia. In addition, evidence for association of *KCNJ11* DNA variants with Type 2 diabetes has been evaluated in multiple studies, and until recently these data have been conflicting. Several recent studies have, however, suggested a role for the aminoacid variant E23K in Type 2 diabetes susceptibility (Hani et al. 1998; Gloyn et al. 2001, 2003; Schwanstecher and Schwanstecher 2002; Love-Gregory et al. 2003; Nielsen et al. 2003). In total we tested four SNPs at the *KCNJ11* locus for association with disease status; of these, three were tightly linked (data not shown) and all three had a statistically significant association with disease status (see Table 2). In our study we replicated the effect of the E23K polymorphism in Type 2 diabetes predisposition (KK homozygous, OR 1.49, *p* = 0.0333; see Table 2) with an OR estimate in agreement with that demonstrated by the meta-analysis of Nielsen et al. (2003); in addition, two other *KCNJ11* SNPs associated with disease risk (SNP74 and SNP76). The recent evidence from multiple studies and from meta-analysis for association of the E23K SNP with Type 2 diabetes, along with in vitro studies using cell lines expressing the E23K mutation showing an increased stimulation threshold of insulinsecretion (Schwanstecher et al. 2002), suggests that E23K is the functional variant leading to increased disease risk. Given our finding of high levels of linkage disequilibrium (LD) between SNP74 and SNP76 with E23K (data not shown), we adjusted the measures of association at these sites for the E23K genotype. These data suggest that these SNPs are independently associated with diabetes (Table 4).

**Table 4:**
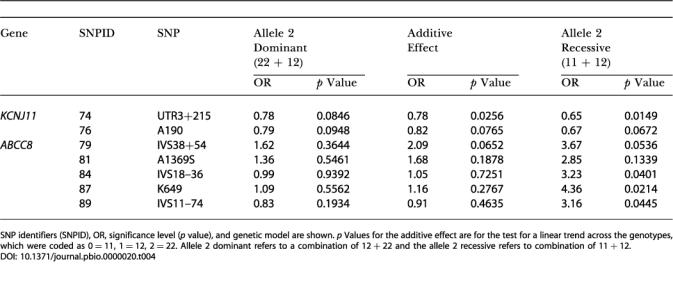
Association of *KCNJ11* and *ABCC8* Variants with Type 2 Diabetes Status Adjusted for E23K Genotype

SNP identifiers (SNPID), OR, significance level (*p* value), and genetic model are shown. *p* Values for the additive effect are for the test for a linear trend across the genotypes, which were coded as 0 = 11, 1 = 12, 2 = 22. Allele 2 dominant refers to a combination of 12 + 22 and the allele 2 recessive refers to combination of 11 + 12

*ABCC8* variants have been associated with Type 2 diabetes in multiple studies (Inoue et al. 1996; T. Hansen et al. 1998; Hart et al. 1999b). However, a recent large study failed to replicate previous associations with Type 2 diabetes (Altshuler et al. 2000). In our study we found evidence for association with Type 2 diabetes in five of 16 *ABCC8* SNPs tested. Owing to the physical mapping of *ABCC8* in close proximity to *KCNJ11*, we further investigated whether the associations at the *ABCC8* locus could be completely explained through LD between *ABCC8* SNPs and the E23K variant at *KCNJ11*. After adjustment for E23K, two *ABCC8* SNPs (SNP79 and SNP81) that were significantly associated with diabetes (*p* = 0.0043 and *p* = 0.0048 for the recessive model; see Table 2) prior to adjustment were no longer significantly associated with diabetes (*p* = 0.0536 and *p* = 0.1339 for the recessive model; see Table 4). However, for the remaining three SNPs (SNP84, SNP87, and SNP89), although the significance levels were reduced, they remained statistically significant (*p* = 0.0401, *p* = 0.0214, and *p* = 0.0445 for the recessive model; see Table 4). Moreover, the OR for two of these SNPs increased to 4.36 and 3.16, respectively. This suggests that there are effects at the *ABCC8* locus that are independent from the E23K *KCNJ11* variant. The lowered significance levels are likely due to loss of power resulting from the adjustment. Our data and that from at least nine other independent association and linkage studies (T. Hansen et al. 2001) have shown some evidence for *ABCC8* involvement in Type 2 diabetes and related phenotypes.

#### *SLC2A2* (encoding GLUT2).

*SLC2A2* encodes the glucose transporter GLUT2, a member of the facilitative glucose transporter family that is highly expressed in pancreatic β-cells and liver. We typed six SNPs in *SLC2A2*, three of which (SNP21, SNP23, and SNP24)****were significantly associated with diabetes status with an OR of approximately 1.4–1.5 (see Table 2). The most highly significant association was with a T110I substitution (OR 1.49, *p* = 0.0059) under a dominant model for the minor allele. In the reduction process prior to haplotype estimations (see Materials and Methods), only one SNP (SNP21) contributed significantly to disease association. Therefore, haplotype reconstructions were not performed. In the QT study, all three disease-associated SNPs were also associated with lower levels of fasting plasma insulin. Rather surprisingly allele 2 (A) at T198, which was associated with increased disease risk, was associated with lower 2-h plasma glucose. No other significant associations with intermediate phenotypes were seen. ****

Multiple previous studies have sought evidence for association or linkage between *SLC2A2* variants and Type 2 diabetes, and most have reported negative results. However, all studies have been small and were insufficiently powered to detect effects of modest size (Li et al. 1991; Baroni et al. 1992; Tanizawa et al. 1994; Moller et al. 2001). *SLC2A2* is a highly plausible candidate gene for Type 2 diabetes, as it is a high K_m_ transporter that regulates entry of glucose into the pancreatic β-cell, thus initiating the cascade of events leading to insulin secretion. GLUT2 is also highly expressed in the liver, where it is involved in the regulation of both glucose uptake and output. It is notable that the alleles that associated with increased diabetes risk were also all associated with lower fasting insulin levels, suggesting that these may influence basal insulin secretion. However, interpretation is complex, as (1) fasting insulin is strongly influenced by insulin sensitivity and (2) the potential risk alleles were not associated with any impairment of insulin secretion in response to a glucose load. Finally, allele 2 (A) at T198, which associated with increased risk of diabetes in the case-control study, was associated with **lower** 2-h glucose in the QT study. Clearly, more detailed genetic mapping combined with functional studies (which will be challenging in humans owing to the inaccessibility of the pancreatic β-cell) will be needed to identify the mechanism whereby variants in this gene influence diabetes risk.

#### *HNF4A* (encoding hepatic nucleotide factor 4α).

*HNF4A* (the *MODY1* gene) encodes an orphan hormone nuclear receptor that, together with *TCF1* (LocusLink ID 6927), encoding HNF1α, *TCF2* (LocusLink ID 6928), encoding HNF1β, and *FOXA2* (LocusLink ID 3170), encoding HNF3β, constitutes part of a network of transcription factors controlling gene expression in pancreatic β-cells, liver, and other tissues. In β-cells, these transcription factors regulate expression of the insulin gene as well as genes encoding proteins involved in glucose transport and metabolism and in mitochondrial metabolism, all of which are linked to insulin secretion (Fajans et al. 2001). While no individual SNP in *HNF4A* was significantly associated with disease status, we identified a haplotype (haplotype B in Figure 3B) that was significantly associated with reduced disease risk (OR 0.83, 95% CI [0.68, 1.00]; data not shown). In the QT study, this ‘reduced-risk' haplotype was****significantly associated with increased insulin secretion (mean = 31.5 pmol/mmol, 95% CI [29.9, 33.3] versus 29.3 pmol/mmol, 95% CI [28.0, 30.6] for haplotype A and 30.9 pmol/mmol, 95% CI [28.1, 34.0] for haplotype C]. Carriers of this haplotype also showed a trend towards lower fasting and 2-h plasma glucose, compared to the subjects with the other haplotypes (see Table 3). *HNF4A* maps to Chromosome 20 (Argyrokastritis et al. 1997) in a region that has been linked to Type 2 diabetes in multiple studies (Bowden et al. 1997; Ji et al. 1997; Zouali et al. 1997; Ghosh et al. 1999; Klupa et al. 2000; Permutt et al. 2001). This positional information, combined with the known role of major mutations at this gene in the causation of autosomal-dominant maturity-onset diabetes of the young (MODY), has led to *HNF4A* being considered as a strong candidate for involvement in Type 2 diabetes. However, most studies to date have failed to identify an association between variants at this locus and disease susceptibility (Moller et al. 1997; Malecki et al. 1998; Ghosh et al. 1999; Price et al. 2000). This study differs from all other previous reports in its examination of haplotypes, as well as in the fact that it included several SNPs not previously examined. Our findings lead us to speculate as to how a particular *HNF4A* haplotype might be associated with lower risk of diabetes and increased insulin secretory capacity. The fact that a multiplicity of heterozygous nonsense and missense mutations in HNF4α lead to an insulinopaenic form of MODY strongly suggests that β-cell dysfunction is sensitive to the amount of HNF4α in the β-cell and that haploinsufficiency is the likely mode of molecular pathogenesis in that condition (Stoffel and Duncan 1997; Shih et al. 2000). It is plausible, therefore, that variants in this gene that enhance expression levels of the protein might lead to increased insulin secretory capacity and protection against diabetes.

#### *INS* (encoding insulin).

The**** *INS* (LocusLink ID 3630) gene encodes the hormone preproinsulin, which upon proteolytic cleavage generates mature insulin and C-peptide. We tested for association of a single SNP in the 3′-UTR (SNP72) of the insulin gene with disease status. This SNP was significantly associated with increased Type 2 diabetes risk under a recessive model for the T** allele (OR 2.02, *p* = 0.0258) (see Table 2). In the QT study this SNP did not associate with any of the intermediate phenotypes studied. The insulin gene variable number tandem repeat (*INS*–VNTR) has been extensively studied and is proposed to exert pleiotropic effects on birth weight and diabetes susceptibility (Huxtable et al. 2000). However, evidence for this has been conflicting and a role for *INS* in Type 2 diabetes predisposition has not been definitively established. The data for the single SNP we tested suggest that either the insulin gene or other loci in LD may be involved in Type 2 diabetes risk.

### Genes Primarily Affecting Insulin Action

#### *INSR* (encoding the insulin receptor).

At the *INSR* locus of the seven SNPs genotyped, we detected a single intronic SNP (SNP131) that was significantly associated with increased disease risk (OR 1.48, *p* = 0.0039 for the dominant model for allele 2) (see Table 2). In the QT study, this SNP also had a nonsignificant association with increased 2-h glucose under a dominant model for allele 2 (see Table S2). Haplotype C (see Figure 3C) for *INSR* was associated with increased disease risk (1.34 mmol/l, 95% CI [1.05, 1.71]; data not shown); in the QT study, there was a nonsignificant trend for subjects carrying this haplotype to have increased values for fasting glucose (5.32 mmol/l, 95% CI [5.24, 5.39] versus 5.27 mmol/l, 95% CI [5.21,5.33] for haplotype B and 5.27 mmol/l, 95% CI [5.23, 5.32] for haplotype A), 2-h glucose (6.00 mmol/l, 95% CI [5.80, 6. 20] versus 5.78 mmol/l, 95% CI [5.61, 5.96] for haplotype B and 5.87 mmol/l, 95% CI [5.75, 5.99] for haplotype A), and fasting insulin (41.8 pmol/l, 95% CI [39.2, 44.5] versus 40.4 pmol/l, 95% CI [38.4, 42.5] for haplotype B and 41.3 pmol/l, 95% CI [39.6, 43.1] for haplotype A) (see Table 3). A role for *INSR* in Type 2 diabetes and related phenotypes has long been sought. Many studies initiated over the past decade have explored the possibility that DNA variants at this locus would not only cause rare syndromes of extreme insulin resistance, but would also associate with increased Type 2 diabetes risk. In particular, the role of the Val985Met in disease predisposition has been analysed in many different populations, but the data remain inconclusive, with some studies suggesting a role for this variant (Hart et al. 1996, 1999b), while others do not support this finding (O'Rahilly et al. 1991, 1992; L. Hansen et al. 1997). In this study we provide preliminary evidence for a role of *INSR* in diabetes susceptibility through genotyping of a previously untested SNP in case-control studies and via haplotype analysis using multiple SNPs in the gene.

#### *PIK3R1* and *SOS1*.

The gene *PIK3R1,* encoding the p85α regulatory subunit of the phosphatidylinositol 3-kinase, is a logical candidate gene for involvement in the development of Type 2 diabetes owing to its role in insulin signal transduction. An intronic variant, SNP42, was associated with increased disease risk under two genetic models (OR 1.41, *p* = 0.0090 for the allele 2 dominant and OR1.34, *p* = 0.0088 for the additive model; see Table 2). In the QT study, SNP42 was significantly associated with increased BMI and showed a borderline significance with increased fasting insulin (measure of insulin resistance) under a dominant model for allele 2 (see Table S2). Obesity is a major risk factor for insulin resistance, and the observed increase in BMI coupled with increased insulin resistance in carriers of allele G at SNP42 suggests that variation at this gene may be increasing Type 2 diabetes risk through impaired insulin action. Other association studies at this locus have focussed on investigating the Met326Ile variant in disease predisposition, with mostly negative results (T. Hansen et al. 1997, 2001; Kawanishi et al. 1997). One study did describe an association with disease status and with QTs underlying Type 2 diabetes (Baier et al. 1998). However, functional data for this polymorphism have suggested that the Ile326 variant may have only minor impact on signalling events (Baynes et al. 2000; Almind et al. 2002). Our data suggest that variation in this gene is a risk factor for the development of Type 2 diabetes, although further detailed studies will be required to elucidate the precise functional variants and mechanisms that lead to increased disease risk.

The gene *SOS1* (*son of sevenless homolog 1* in Drosophila) encodes a guanine nucleotide exchange factor that functions in the transduction of signals that control cell growth and differentiation. We analysed two SNPs for association with disease status, a nonsynonymous SNP (N1011S) and an intronic variant (SNP8). While the nonsynonymous S1011 variant, which was very rare (minor allele, 0.003), did not associate with disease status, the intronic SNP was highly significantly associated with decreased disease risk (OR 0.58, *p* = 0.0032) (see Table 2), despite not showing any effects in the QT study. To our knowledge, this is the first investigation into the role of *SOS1* in Type 2 diabetes risk. The data presented here suggest that further investigation into the potential role of common variants at this gene and diabetes risk is warranted.

### Other Genes

*PPARGC1* (LocusLink ID 10891) encodes a transcriptional coactivator of nuclear receptors with a critical role in regulating multiple aspects of energy metabolism, including adaptive thermogenesis (Puigserver et al. 1998), mitochondrial biogenesis (Wu et al. 1999), fatty acid β-oxidation (Vega et al. 2000), the control of hepatic gluconeogenesis (Herzig et al. 2001; Yoon et al. 2001), and the control of glucose uptake (Michael et al. 2001). *PPARGC1* SNP30 (Thr528Thr), which was associated with decreased disease risk (see Table 2), was rather surprisingly associated with decreased insulin secretion in the QT study (see Table S2). In this locus, Thr528Thr has not been previously associated with diabetes, and our data most likely reflect stochastic variation at this site. The Gly482Ser has in some studies been shown to be associated with increased Type 2 diabetes risk (Ek et al. 2001), but not in others (Hara et al. 2002; Lacquemant et al. 2002; Muller et al. 2003), and has additionally been associated with insulin resistance (Hara et al. 2002), obesity indices in women (Esterbauer et al. 2002), and mean insulin secretory response and lipid oxidation (Muller et al. 2003). In our study, this allele was not associated with increased diabetes risk, but rather was associated with a lower risk of diabetes under a recessive model (OR 0.67, *p* = 0.0295) (see Table 2). The opposing results for this polymorphism and the fact that the amino acid change Gly482Ser is unlikely to be a major functional change (Esterbauer et al. 2002) may indicate that the contributing functional polymorphism may be an unidentified variant in LD with the Gly482Ser. ****

Amongst the remaining genes tested, of particular interest are the results observed in *PYY* (encoding polypeptide YY; LocusLink ID 5697). An intronic variant, IVS3+68, showed a significant association with increased Type 2 diabetes risk under two genetic models (OR 1.47, *p* = 0.0240 in the allele 2 dominant; OR 1.47, *p* = 0.0157 in the additive effect allele 2) (see Table 2), but no evidence of association with underlying traits was observed in the QT study. Early functional studies suggested an inhibitory role of *PYY* in glucose-stimulated insulin secretion (Bertrand et al. 1992; Nieuwenhuizen et al. 1994), which led us to evaluate the potential role of variants at this gene in Type 2 diabetes predisposition. Recent data have shown that the peptide PYY_3–36_ encoded by this gene inhibits food intake and reduces weight gain when injected in rats, while physiological infusions of PYY_3–36 _in humans decreased food intake by 33% (Batterham et al. 2002). Although our data do not show an association between the intronic variant SNP122 with BMI, they suggest a putative role for *PYY* in Type 2 diabetes predisposition. As we only tested a noncoding variant in *PYY*, it is possible that the association is due to other contributing variants within the gene and that a link between those and BMI is still plausible.

In the genes *ABCC9* (LocusLink ID 10060)** and *LIPC* (LocusLink ID 3990), single SNPs of modest significance were associated with disease status in the case control and therefore are not discussed further here.

### Examination of Other Previously Reported Associations

We were unable to confirm some associations observed in other studies. The *PPARG* Pro12Ala Pro allele has previously been shown to confer susceptibility to Type 2 diabetes, with the Ala allele providing a decreased risk (Altshuler et al. 2000). Our results for this polymorphism show the same direction and magnitude of effect for Ala/Ala versus Ala/Pro and Pro/Pro genotypes (OR 0.54; derived from data in Table 2), but the association was not statistically significant (*p* = 0.2269). The lower limit of the 95% CI for the protective effect of the Ala allele (OR 1.08, 95% CI [0.82,1.42], *p* = 0.583) is still consistent with the results of the meta-analysis by Altshuler et al. (2000). Our study was not sufficiently powered to detect the small effects expected for the predisposing Pro allele. The study only had 25.4% power to detect an OR of 1.25 for the Pro allele that occurred in 89.4% of our control population. It is also possible that the Pro12Ala variant does not affect diabetes susceptibility in this population, because of the dependence of the allele effect on environmental factors such as dietary fat composition (Luan et al. 2001).

In the *ENPP1* (LocusLink ID 5167)** gene** (commonly known as *PC-1*) the K121Q polymorphism has variably been found to be both associated with increased Type 2 diabetes risk (Gu et al. 2000; Rasmussen et al. 2000; Hegele et al. 2001) and with insulin resistance QTs (Pizzuti et al. 1999; Gu et al. 2000; Rasmussen et al. 2000). In our study we did not find evidence for association between the K121Q polymorphism and Type 2 diabetes (OR 1.10, *p* = 0.5277 for the dominant model for allele 2; OR 1.07, *p* = 0.6290 for the additive effect for allele 2; OR 0.86, *p* = *0.7*496 for a recessive model for allele 2; data derived from** Table 2). Analysis of this allele in our QT study showed that QQ individuals have higher mean BMI levels compared to carriers of the K121 allele (28.3 kg/m^2^ [26.4, 30.2] versus 26.1 [25.6, 26.7] in KQ subjects, 26.3 [25.9, 26.6] in KK subjects; data not shown). *PPP1R3A* (protein phosphatase 1 regulatory subunit 3; LocusLink ID 5506), which encodes the muscle-specific regulatory subunit of PP1, has been investigated as a potential diabetogene. Evidence for a role of the *PPP1R3A* D905Y polymorphism in Type 2 diabetes risk has also been conflicting (L. Hansen et al. 1995, 2000; Hegele et al. 1998; Xia et al. 1998). While in this study we did not find an association between the D905Y variant and disease risk, we have previously described an association between a rare frameshift and premature stop variant with Type 2 diabetes risk under a dominant model (OR 5.03, *p* = 0.0110) in this population (Savage et al. 2002).

## Concluding Remarks

This study, which to our knowledge is the largest of its kind yet reported in Type 2 diabetes, has provided evidence for the existence of variants in certain key candidate genes that influence the risk of Type 2 diabetes and, in some cases, has afforded clues as to the pathophysiological mechanism whereby those effects on disease risk might be mediated. By its very nature, any study of candidate genes, however large, is restricted in scope, and it is likely that other variants (namely in regulatory regions, which we did not cover) in the genes that we have considered, as well as ones that we have not, may exist and have effects equal to or greater than those we have demonstrated. In addition, this study is not intended to be an ‘exclusion study,' as many issues that relate to coverage of any given gene, environmental risk factors, and power in our populations do not allow us to definitively assert that negative findings correspond to genes that truly do not play a role in Type 2 diabetes predisposition.

**Figure 4:**
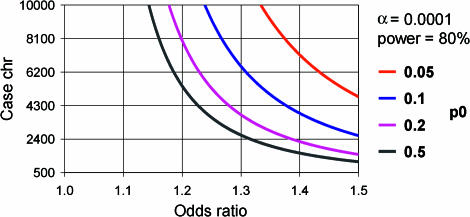
Size of Case-Control Study Required to Detect Small Risk Effects The number is shown of the case chromosomes (assuming an equal number of control chromosomes) required to attain 80% power to detect associations with the OR varying between 1.0 and 1.5 and with a Type 1 error rate of 0.01%. Abbreviations: p0, frequency of the predisposing allele; chr, number of chromosomes. Graphs were plotted with the PS power and sample-size program (DuPont and Plummer 1997).

The power of our study to detect small effects in uncommon variants was low. Evidence from many recent studies now suggests that in Type 2 diabetes the effects are likely in the range of OR 1.15–1.5. It is clear that much larger studies than that reported here are required for such effects (Figure 4), in particular when adjusting to a lower Type 1 error rate of 0.01% to compensate for multiple testing. The significance of the associations we report have been described without adjustment for the number of tests undertaken, and thus the group of positive associations is likely to contain a proportion that is falsely positive. There is no consensus about the ideal method for adjusting the probability of an observation occurring by chance for multiple testing. The simple Bonferroni correction would constitute overadjustment because the 152 genetic markers in this study are not independent. In addition, in the false-discovery rate method (Benjamini and Hochberg 1995), it is assumed that all N tests are carried out simultaneously, which may not correspond to reality if groups genotype one set of SNPs, as in this study, but then report results for additional SNPs at a later date. It is not clear whether the number of tests N should reflect the number to date or the number one might potentially undertake by continuing working through projects like these. An alternative Bayesian approach leading to a ‘genome-wide’ significance level for association, such as has been done for whole-genome linkage studies (Lander and Kruglyak 1995), might be preferable. However, this also runs into difficulties. In studies that are not based on fine-mapping of linkage intervals, but rather on candidate genes selected on the basis of data from other studies, including previous reports of association, it is unclear what level of prior probability of association should be used. As a result of this uncertainty about the appropriate method of correction for multiple testing, our preferred strategy is to report the number of tests done and to encourage readers to interpret the significance tests in that light, acknowledging that the results will require replication in other cohorts.

Although we have not undertaken a formal replication of the case-control study, we have used a complimentary QT population to examine the association of the variants studied with continuously distributed measures of glucose tolerance, insulin secretion, and insulin action. This provides different information to a replication case-control study, as it may identify pathophysiological mechanisms by which the association with diabetes arises. We are cautious about putting forward particular variants as established ‘diabetogenes' and enthusiastically invite researchers to examine these candidate variants in their own particular populations. Indeed, as the genetic architecture of Type 2 diabetes may vary between populations, it is critical that such variants are examined in multiple diverse ethnic groups. As with the Pro12Ala *PPARG* example, it is likely that meta-analysis of several studies will be required to narrow the CIs around the point estimates of association seen in any single study. This will be especially important when the association is weak, as it is for Pro12Ala, because few individual case-control studies, including the one reported here, are currently powered to detect very small increases in risk. It is, however, important that such meta-analyses include all studies of variants examined, rather than only those that are individually published, to avoid publication bias.

The associations we describe are highly biologically plausible and in many of the genes are supported by associations with multiple SNPs at the same locus. These include genes affecting both insulin secretion and insulin action. Given the importance of both insulin resistance and defective β-cell function to the pathogenesis of Type 2 diabetes, it is intriguing that we have found a disproportionate representation of genes affecting pancreatic β-cell function among those that were found to be associated with diabetes risk. This contrasts with the impact of known environmental factors and their correlates (e.g., high-fat diet, lower physical activity, obesity, and central fat distribution), all of which are thought to have their major influence on diabetes risk through impairment of insulin action. While it would be premature to put forward any definitive model for the causation of Type 2 diabetes, it is tempting to speculate that the ‘insulin resistance' component of the disease may have a substantial environmental influence modulated by polygenic effects, some of which may relate to molecules identified in this and other studies. On the other hand, the ability of the pancreatic β-cell to continue to secrete sufficient insulin to maintain life-long normoglycaemia may be more profoundly influenced by genetic factors, some of which are reported herein. It will be critical to examine the functional consequences of such variants, a task that will be particularly challenging when it comes to genes influencing human β-cell function, as it is entirely possible that this disproportionate representation of β-cell genes may be a reflection of our success in choosing diabetes genes in each of the candidate genes in the major groupings.

The success of the approach presented here, although necessarily limited in scope, suggests that the****systematic examination of panels of biological candidate genes in large, well-characterised populations may usefully complement positional approaches to the identification of allelic variants conferring susceptibility to complex polygenic disease. The detection of multiple small gene effects accentuates the need for larger populations in order to reliably identify the types of effects (OR 1.15–1.5) we now expect for complex diseases.

## Materials and Methods

### 

#### Methods for SNP discovery and SNP selection for genotyping.

SNP discovery was performed by a high-fSSCP-based analysis, as previously described (Thorpe et al. 1999). Genomic structure was determined for all genes, and primers were designed to span the exons and splice junctions. To detect common variants, genes were screened against one or more of a variety of different DNA panels, which included a 47-member multiethnic human diversity panel (comprised of 17 Europid, seven Hispanic, 13 East Asian, and ten African-American subjects), our 129-member severe insulin-resistant cohort (Barroso et al. 1999), a panel of 47 European-American samples, a panel of 47 African-American samples, and in some cases a panel of 94 samples (half European and half Asian Indian). Some genes had only partially screened coding sequence and splice junctions at the time of SNP selection for genotyping. In addition, we had access to an internal database of in silico SNPs that had been validated against 100 samples. Choice of polymorphisms for further testing in association studies was not constrained by the type of variant (e.g., nonsynonymous, silent, noncoding), although higher priority was given to variants with a likely effect on protein structure and function. Polymorphisms with a minor allele frequency greater than or equal to 5% were selected for further testing in population-based studies. In some instances, polymorphisms of lower allele frequency were genotyped to examine whether lower frequency variants with high penetrance might account for some cases of polygenic disease. Polymorphisms of lower frequency were also genotyped when, at the time of selection for genotyping, no other variants of known frequency were identified in the gene to test.

#### Populations for SNP genotyping.

The Cambridgeshire Case-Control Population (Poulton et al. 2002; Halsall et al. 2003) consists of a collection of 517 Type 2 diabetics and 517 matched controls. The cases were a random sample of Europid men and women with Type 2 diabetes, aged 47–75 years, from a population-based diabetes register in a geographically defined region in Cambridgeshire, United Kingdom. The presence of Type 2 diabetes in these subjects was defined as onset of diabetes after the age of 30 years without use of insulin therapy in the first year after diagnosis. The control subjects were individually age-, gender-, and geographical location-matched to each of the cases. Controls were not matched by BMI to cases. Potential controls that had HbA1c levels greater than 6% were excluded, as this group may contain a higher proportion of individuals with previously undiagnosed diabetics*.* HbA1c was assayed using high performance liquid chromatography on a BioRad Diamat 33 (Hercules, California, United States), according to the method of Standing and Taylor (1992). The coefficient of variation (CV) was 3.6% at the lower end of the range (mean = 4.94%) and 3.0% at the upper end (mean = 9.76%). Further details on the characteristics of the subjects are shown in Table 5. ****

**Table 5:**
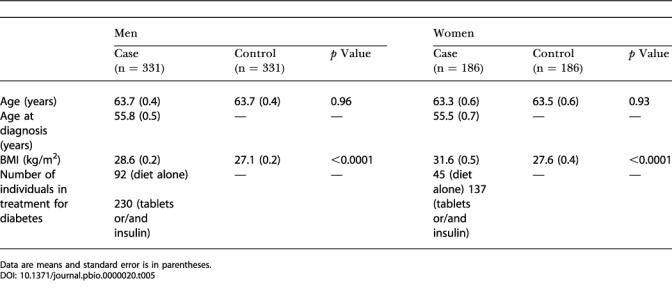
Study Subjects in the Cambridgeshire Case-Control Study

Data are means and standard error is in parentheses

The** QT study population is a collection of 1,100 samples collected for the Ely Study, a prospective population-based cohort study of the aetiology and pathogenesis of Type 2 diabetes and related metabolic disorders (Wareham et al. 1999). Height was measured using rigid stadiometer, and weight was measured on Seca-calibrated scales with participants in light clothing. BMI was estimated as weight (kg) divided by height (m) squared. Plasma glucose was measured in the routine National Health Service Laboratory at Addenbrooke's Hospital, using the hexokinase method (Kunst et al. 1983). Plasma insulin was measured by two-site immunometric assays with either ^125^I or alkaline phosphatase labels (Sobey et al. 1989; Alpha et al. 1992). Cross-reactivity with intact proinsulin was less than 0.2% and CVs were less than 7%.

#### Methods for genotyping.

Genotyping was performed using an adaptation of the fluorescence polarisation template-directed incorporation (FP-TDI) method described by Chen et al.(1999). In short, PEP-amplified DNA samples were PCR-amplified in 8 μl reactions with primers flanking the variant site; unincorporated dNTP and remaining unused primer were degraded by exonuclease I and shrimp alkaline phosphatase at 37°C for 45 min before the enzymes were heat-inactivated at 95°C for 15 min. At the end of the reaction, the samples were held at 4°C. Single base primer extension reactions were performed as previously described (Chen et al. 1999), and allele detection was performed by measuring fluorescence polarisation on an LJL Analyst fluorescent reader (Molecular Devices, Sunnyvale, California, United States). The PEP protocol was specifically developed and tested to ensure that allele bias was not introduced during the amplification process. A minimum of 12% internal replicate samples within each population (case control and QT) were included in all genotyping tests to assess genotyping accuracy. Only assays that provided 100% concordance between replicates were analysed for association. The genotyping pass rate was greater than 90% once a working assay had been established. There was an 85% success rate for an SNP to be converted into a working assay at the first attempt, with a number of failed assays recovered by designing an assay to the reverse strand. ****

#### Statistical analysis.

All analyses used SAS 8.02 (SAS Institute, Cary, North Carolina, United States) or Stata 7.0 (Stata Corporation, College Station, Texas, United States) statistical programs, unless otherwise stated. Agreement with Hardy–Weinberg equilibrium was tested using a χ^2^ ‘goodness-of-fit' test. The disequilibrium coefficient for the controls (*r^2^*) was calculated (Lewontin 1964).

For the case-control study, tests for association with disease status under dominant, additive, and recessive models were undertaken using univariate logistic regression analysis. Dominance was defined in terms of allele 2 effects; in the dominant allele 2 model, homozygous subjects for allele 1 were compared with carriers of allele 2; in the recessive allele 2 model, carriers of allele 1 were compared with homozygous subjects for allele 2. In some cases, a large number of polymorphisms within a gene were typed. To reduce complexity, a subset of markers within a gene associated with diabetes status was identified using backward logistic regression. Any polymorphism that had a *p* value greater than 0.1 was removed from the model. The genotypes were assumed as having additive effects. *p* values for the additive effect are for the test for a linear trend across the genotypes, which were coded as 0 = 11, 1 = 12, 2 = 22. Where the subset consisted of more than one polymorphism within a gene, haplotype analysis was performed. To avoid possible errors due to either genotyping or the estimation process, only haplotypes that had a frequency greater than 5% were considered for further analysis. Haplotype frequencies were estimated using maximum-likelihood methods. A log-linear model embedded with the expectation-maximization algorithm was fitted to a frequency table (Chiano and Clayton 1998; Mander 2001). Differences in haplotype distributions between the diabetic and nondiabetic groups were examined using a likelihood-ratio statistic (Mander 2001). To obtain separate ORs for each haplotype, the most common haplotype was used as the reference category. CIs were obtained using a profile-likelihood approach (Mander 2001).

For the QT study, the distributions of fasting plasma glucose, 2-h plasma glucose, fasting plasma insulin, and insulin increment were skewed and were thus normalised by logarithmic transformation. Baseline and follow-up measurements of BMI, fasting and 2-h plasma glucose, fasting plasma insulin, and 30-min insulin increment during an oral glucose tolerance test were collected. Where two measures were available, the mean was used. Otherwise, a single measure (either baseline or follow-up) was used for further analysis. The subset of SNPs identified in the case-control study was used. In separate dominant, additive, and recessive models, adjusting for age and sex, genotype differences in these measurements were modelled using the General Linear Model procedure in the statistical package SAS. For each individual, a list of possible haplotypes and their probabilities was obtained using Snphap software (http://www-gene.cimr.cam.ac.uk/clayton/software/). Haplotypes with a frequency greater than 5% were the same as those reconstructed in the case-control study. Only haplotypes that had a frequency greater than 5% and individuals that had at least one marker typed were considered for analysis. As currently haplotype analysis software cannot handle repeated measurements, the average of two measurements was used for further analysis. Associations of haplotypes (adjusted for age and sex) with the QTs were determined by cluster-weighted regression analysis, thereby taking into account nonindependent multiple observations from an individual (Huber 1967; White 1980, 1982). QT means and their 95% CI were estimated for each haplotype.

## Supporting Information

Table S1 Genotype Counts and Frequencies for All SNPs Genotyped in This Study (79 KB XLS).

Table S2 Single SNP Associations with QTs (174 KB DOC).

### Accession Numbers

The LocusLink accession numbers discussed in this paper are 3170, 3172, 3630, 3643, 3767, 3990, 5167, 5295, 5468, 5506, 5697, 6514, 6654, 6833, 6927, 6928, 10060, 10891, and 11132.

## Abbreviations

ABC: ATP-binding cassette
BMI: body mass index
CI: confidence interval
CV: coefficient of variation
fSSCP: fluorescent single-stranded conformation polymorphism
HbA1c: glycated haemoglobin
INS0: fasting insulin
IVS: intervening sequence
LD: linkage disequilibrium
MODY: maturity-onset diabetes of the young
OR: odds ratio
PG0: fasting plasma glucose levels
PHHI: persistent hyperinsulinaemia hypoglycaemia of infancy
PPARG: peroxisome proliferator-activated receptor γ
PP1: protein phosphatase 1
QT: quantitative trait
SNP: single nucleotide polymorphism
VNTR: variable number tandem repeat.
