# In PLoS Biology, volume 1, issue 1: Candidate Gene Association Study in Type 2 Diabetes Indicates a Role for Genes Involved in β-Cell Function as Well as Insulin Action

**DOI:** 10.1371/journal.pbio.0000092

**Published:** 2003-12-22

**Authors:** Inês Barroso, Jian'an Luan, Rita P. S Middelberg, Anne-Helen Harding, Paul W Franks, Rupert W Jakes, David Clayton, Alan J Schafer, Stephen O'Rahilly, Nicholas J Wareham

One of the variants associated with increased diabetes risk was incorrectly indicated throughout this article. The A1369S variant in the gene ABCCB should have been written S1369A. The alanine variant is associated with increased risk. This mistake affects Tables 2 and 4, the text of the article in the section entitled "*ABCCB* and *KCNJ11*" on page 45, and the Supporting Information Tables S1 and S2.

The full text XML and HTML versions of the article, and the supporting Tables S1 and S2 have been corrected online.

