# Supplementary material for: Candidate Gene Association Study in Type 2 Diabetes Indicates a Role for Genes Involved in β-Cell Function as Well as Insulin Action
Source: PLoS Biol. 2003 Oct 13;1(1):e20. doi: 10.1371/journal.pbio.0000020 (PMC212698; doi:10.1371/journal.pbio.0000020)
Supplement: Table S2 — (174 KB DOC). [file pbio.0000020.st002.doc]

| **Table S2. Single SNP Associations with Quantitative Traits.**  Age and sex-adjusted means (not adjusted for BMI) for each genotype at individual SNPs significantly associated with disease in the case-control study. Associations significant at 0.10 and below are in ***italics bold***, with association 0.05 or below in **red** **bold**. For genes with multiple SNPs tested, backward logistic regression was performed to select SNPs significantly associated with disease (see methods section). When more than one SNP remained in the model these SNPs were used in haplotype reconstructions. Often only one SNP remained in the model and these are marked with an asterisk (*). BMI-body mass index (kg/m^2^), PG0-fasting plasma glucose (mmol/l), 2hPG-2 hrs plasma glucose (mmol/l), INS0-fasting insulin (pmol/l), Ins inc-30 min insulin increment (pmol/mmol). The means for PG0, 2hPG, INS0 and Ins inc are geometric means. p-values for the additive model are for the test for a linear trend across the genotypes which were coded as 0="11", 1="12", 2="22". Dominant refers to combination of 12+22 and recessive refers to combination of 11+12. | | | | | | | | |
| --- | --- | --- | --- | --- | --- | --- | --- | --- |
| SNP (SNP ID) and |  | 11 | 12 | 22 | significance in model (p-value) | | |  |
| number of individuals with each genotype | Trait | Mean (95% CI) | Mean (95% CI) | Mean (95% CI) | dominant | additive | recessive |  |
| SOS1 | BMI | 26.2 (25.9, 26.5) | 26.6 (25.8, 27.4) | 24.7 (21.3, 28.1) | 0.550 | 0.734 |  |  |
| IVS17+53 (SNP8)* | PG0 | 5.29 (5.25, 5.34) | 5.33 (5.21, 5.45) | 5.17 (4.73, 5.65) | 0.681 | 0.798 |  |  |
| n=799/99/6 | 2hPG | 5.86 (5.74, 5.97) | 6.00 (5.68, 6.33) | 5.38 (4.32, 6.70) | 0.533 | 0.694 |  |  |
|  | INS0 | 41.3 (39.7, 42.9) | 41.0 (36.9, 45.6) | 41.4 (26.9, 63.8) | 0.927 | 0.936 |  |  |
|  | Ins inc | 29.8 (28.6, 31.0) | 30.7 (27.4, 34.5) | 35.4 (22.2, 56.5) | 0.509 | 0.439 |  |  |
| SLC2A2 | BMI | 26.4 (26.1, 26.7) | 25.8 (25.3, 26.4) | 26.6 (24.5, 28.6) | 0.126 | 0.196 |  |  |
| IVS5-15 (SNP21)* | PG0 | 5.31 (5.26, 5.35) | 5.28 (5.20, 5.36) | 5.27 (4.99, 5.57) | 0.552 | 0.555 |  |  |
| n=675/205/16 | 2hPG | 5.93 (5.81, 6.05) | 5.77 (5.55, 5.99) | 5.49 (4.80, 6.28) | 0.142 | 0.109 |  |  |
|  | INS0 | 42.2 (40.5, 43.9) | 39.3 (36.5, 42.2) | 34.5 (26.5, 44.9) | ***0.051*** | **0.033** |  |  |
|  | Ins inc | 29.9 (28.6, 31.3) | 29.5 (27.2, 32.0) | 27.8 (20.8, 37.1) | 0.679 | 0.615 |  |  |
| T198 (SNP23) | BMI | 26.4 (26.1, 26.7) | 25.6 (25.0, 26.3) | 27.1 (24.7, 29.5) | ***0.080*** | 0.123 |  |  |
| n=714/167/12 | PG0 | 5.32 (5.28, 5.36) | 5.20 (5.12, 5.29) | 5.38 (5.05, 5.73) | ***0.065*** | *0.072* |  |  |
|  | 2hPG | 5.95 (5.83, 6.07) | 5.64 (5.41, 5.88) | 5.68 (4.86, 6.64) | ***0.076*** | **0.030** |  |  |
|  | INS0 | 42.3 (40.7, 44.0) | 37.7 (34.8, 40.9) | 34.0 (25.2, 46.0) | **0.020** | **0.005** |  |  |
|  | Ins inc | 29.8 (28.5, 31.1) | 30.0 (27.4, 32.8) | 22.4 (16.1, 31.3) | 0.247 | 0.496 |  |  |
| T110I (SNP24) | BMI | 26.4 (26.1, 26.7) | 25.8 (25.2, 26.4) | 26.6 (24.5, 28.6) | ***0.087*** | 0.141 |  |  |
| n=661/212/16 | PG0 | 5.30 (5.26, 5.35) | 5.28 (5.20, 5.36) | 5.28 (5.00, 5.57) | 0.584 | 0.596 |  |  |
|  | 2hPG | 5.91 (5.79, 6.04) | 5.80 (5.59, 6.01) | 5.50 (4.82, 6.28) | 0.263 | 0.201 |  |  |
|  | INS0 | 42.2 (40.5, 44.0) | 39.4 (36.6, 42.3) | 34.6 (26.6, 45.0) | 0.055 | **0.036** |  |  |
|  | Ins inc | 30.1 (28.8, 31.5) | 29.5 (27.2, 31.9) | 27.7 (20.8, 37.0) | 0.588 | 0.528 |  |  |
| PPARGC1 | BMI | 26.5 (26.0, 27.0) | 26.1 (25.8, 26.5) | 26.4 (25.7, 27.1) |  | 0.570 |  |  |
| T528 (30)* | PG0 | 5.33 (5.26, 5.40) | 5.29 (5.24, 5.35) | 5.28 (5.19, 5.37) |  | 0.346 |  |  |
| n=295/445/156 | 2hPG | 5.92 (5.74, 6.11) | 5.86 (5.71, 6.01) | 5.91 (5.67, 6.18) |  | 0.879 |  |  |
|  | INS0 | 43.2 (40.6, 45.9) | 40.4 (38.5, 42.5) | 40.9 (37.6, 44.5) |  | 0.202 |  |  |
|  | Ins inc | 31.5 (29.4, 33.7) | 29.1 (27.6, 30.8) | 28.0 (25.5, 30.7) |  | **0.028** |  |  |
| G482S (31) | BMI | 26.5 (26.1, 27.0) | 26.1 (25.7, 26.5) | 26.2 (25.5, 27.0) |  |  | 0.894 |  |
| n=355/434/111 | PG0 | 5.33 (5.26, 5.39) | 5.29 (5.23, 5.35) | 5.26 (5.15, 5.37) |  |  | 0.461 |  |
|  | 2hPG | 5.92 (5.75, 6.09) | 5.85 (5.70, 6.00) | 5.92 (5.62, 6.23) |  |  | 0.811 |  |
|  | INS0 | 42.1 (39.8, 44.5) | 41.4 (39.3, 43.5) | 38.4 (34.8, 42.5) |  |  | 0.135 |  |
|  | Ins inc | 31.1 (29.2, 33.1) | 29.3 (27.7, 31.0) | 26.9 (24.1, 30.0) |  |  | ***0.061*** |  |
| PIK3R1 | BMI | 26.0 (25.7, 26.4) | 26.8 (26.4, 27.3) | 25.8 (24.6, 27.0) | **0.017** | 0.104 |  |  |
| IVS4+82 (42)* | PG0 | 5.29 (5.24, 5.34) | 5.31 (5.25, 5.38) | 5.32 (5.15, 5.49) | 0.616 | 0.625 |  |  |
| n=552/310/49 | 2hPG | 5.87 (5.74, 6.01) | 5.95 (5.77, 6.13) | 5.56 (5.15, 6.00) | 0.877 | 0.665 |  |  |
|  | INS0 | 40.3 (38.5, 42.1) | 43.6 (41.0, 46.3) | 39.5 (34.0, 45.9) | ***0.071*** | 0.209 |  |  |
|  | Ins inc | 29.7 (28.2, 31.2) | 29.8 (27.9, 31.8) | 31.3 (26.6, 36.9) | 0.796 | 0.656 |  |  |
| INS | BMI | 26.4 (26.0, 26.8) | 26.1 (25.6, 26.5) | 26.6 (25.4, 27.8) |  |  | 0.630 |  |
| 3p+9 (72) | PG0 | 5.27 (5.22, 5.32) | 5.32 (5.26, 5.38) | 5.39 (5.22, 5.56) |  |  | 0.239 |  |
| n=515/325/48 | 2hPG | 5.92 (5.78, 6.06) | 5.84 (5.67, 6.02) | 5.74 (5.31, 6.20) |  |  | 0.526 |  |
|  | INS0 | 41.0 (39.1, 43.0) | 41.8 (39.4, 44.3) | 42.9 (36.8, 50.0) |  |  | 0.639 |  |
|  | Ins inc | 29.9 (28.4, 31.5) | 30.1 (28.2, 32.1) | 28.2 (23.8, 33.3) |  |  | 0.470 |  |
| KCNJ11 | BMI | 26.1 (25.7, 26.6) | 26.2 (25.9, 26.6) | 27.0 (26.2, 27.7) | 0.357 | 0.114 | ***0.071*** |  |
| 3p+215 (74) | PG0 | 5.33 (5.27, 5.39) | 5.27 (5.21, 5.32) | 5.35 (5.24, 5.46) | 0.274 | 0.735 | 0.362 |  |
| n=333/452/114 | 2hPG | 5.96 (5.78, 6.13) | 5.84 (5.69, 5.99) | 5.89 (5.60, 6.19) | 0.336 | 0.477 | 0.985 |  |
|  | INS0 | 41.0 (38.7, 43.5) | 41.3 (39.3, 43.5) | 42.5 (38.5, 46.9) | 0.718 | 0.582 | 0.568 |  |
|  | Ins inc | 29.8 (27.9, 31.7) | 29.9 (28.3, 31.6) | 29.1 (26.1, 32.4) | 0.987 | 0.831 | 0.654 |  |
| A190 (76) | BMI | 26.2 (25.7, 26.6) | 26.3 (25.8, 26.7) | 26.8 (25.9, 27.7) |  | 0.305 | 0.236 |  |
| n=400/413/78 | PG0 | 5.32 (5.26, 5.38) | 5.27 (5.21, 5.33) | 5.35 (5.22, 5.49) |  | 0.749 | 0.407 |  |
|  | 2hPG | 5.94 (5.78, 6.11) | 5.78 (5.62, 5.94) | 6.03 (5.67, 6.41) |  | 0.626 | 0.371 |  |
|  | INS0 | 41.3 (39.2, 43.5) | 40.4 (38.4, 42.6) | 43.8 (38.9, 49.3) |  | 0.749 | 0.270 |  |
|  | Ins inc | 30.2 (28.5, 32.0) | 29.4 (27.7, 31.1) | 28.1 (24.6, 32.0) |  | 0.284 | 0.395 |  |
| E23K (77) | BMI | 26.6 (26.1, 27.0) | 26.1 (25.7, 26.5) | 25.8 (25.0, 26.6) |  |  | 0.252 |  |
| n=350/434/111 | PG0 | 5.30 (5.24, 5.36) | 5.26 (5.20, 5.31) | 5.27 (5.16, 5.38) |  |  | 0.852 |  |
|  | 2hPG | 5.94 (5.77, 6.12) | 5.81 (5.66, 5.96) | 6.01 (5.71, 6.32) |  |  | 0.396 |  |
|  | INS0 | 42.8 (40.5, 45.3) | 39.7 (37.7, 41.7) | 40.4 (36.5, 44.6) |  |  | 0.757 |  |
|  | Ins inc | 30.9 (29.0, 32.8) | 30.0 (28.4, 31.7) | 28.5 (25.6, 31.8) |  |  | 0.280 |  |
| ABCC8 | BMI | 26.6 (26.1, 27.0) | 26.2 (25.8, 26.6) | 25.8 (25.1, 26.6) |  | ***0.080*** | 0.190 |  |
| IVS38+54 (79) | PG0 | 5.34 (5.28, 5.40) | 5.28 (5.22, 5.33) | 5.29 (5.18, 5.40) |  | 0.237 | 0.808 |  |
| n=342/440/115 | 2hPG | 6.00 (5.83, 6.18) | 5.79 (5.64, 5.94) | 6.00 (5.70, 6.31) |  | 0.448 | 0.474 |  |
|  | INS0 | 42.9 (40.5, 45.4) | 40.1 (38.1, 42.2) | 40.6 (36.8, 44.8) |  | 0.151 | 0.758 |  |
|  | Ins inc | 29.9 (28.1, 31.8) | 29.9 (28.3, 31.6) | 28.4 (25.5, 31.6) |  | 0.535 | 0.391 |  |
| A1369S (81) | BMI | 26.5 (26.0, 26.9) | 26.2 (25.8, 26.6) | 25.7 (25.0, 26.5) |  | 0.125 | 0.163 |  |
| n=338/444/111 | PG0 | 5.33 (5.27, 5.40) | 5.28 (5.22, 5.33) | 5.29 (5.18, 5.40) |  | 0.277 | 0.826 |  |
|  | 2hPG | 5.98 (5.80, 6.15) | 5.78 (5.63, 5.93) | 5.94 (5.64, 6.25) |  | 0.400 | 0.653 |  |
|  | INS0 | 43.1 (40.6, 45.6) | 40.1 (38.1, 42.1) | 40.3 (36.5, 44.6) |  | 0.107 | 0.658 |  |
|  | Ins inc | 30.0 (28.2, 32.0) | 29.8 (28.2, 31.5) | 28.6 (25.6, 31.9) |  | 0.530 | 0.461 |  |
| IVS18-36 (84) | BMI | 26.2 (25.9, 26.5) | 26.3 (25.7, 26.8) | 28.2 (26.6, 29.9) |  |  | **0.016** |  |
| n=655/212/26 | PG0 | 5.30 (5.26, 5.35) | 5.27 (5.19, 5.35) | 5.53 (5.29, 5.77) |  |  | ***0.057*** |  |
|  | 2hPG | 5.90 (5.77, 6.02) | 5.82 (5.60, 6.04) | 6.04 (5.44, 6.72) |  |  | 0.611 |  |
|  | INS0 | 41.1 (39.5, 42.9) | 41.2 (38.3, 44.3) | 46.8 (38.1, 57.6) |  |  | 0.224 |  |
|  | Ins inc | 29.5 (28.2, 30.9) | 30.4 (28.1, 32.9) | 28.7 (22.9, 36.0) |  |  | 0.757 |  |
| K649 (87) | BMI | 26.3 (25.9, 26.6) | 26.2 (25.6, 26.8) | 27.0 (25.3, 28.7) |  |  | 0.394 |  |
| n=665/213/23 | PG0 | 5.30 (5.25, 5.34) | 5.29 (5.21, 5.37) | 5.41 (5.17, 5.66) |  |  | 0.376 |  |
|  | 2hPG | 5.88 (5.76, 6.01) | 5.86 (5.64, 6.08) | 5.92 (5.29, 6.62) |  |  | 0.901 |  |
|  | INS0 | 41.1 (39.4, 42.8) | 41.9 (38.9, 45.0) | 41.1 (33.0, 51.3) |  |  | 0.981 |  |
|  | Ins inc | 29.8 (28.4, 31.1) | 30.9 (28.6, 33.4) | 23.6 (18.6, 30.1) |  |  | ***0.054*** |  |
| IVS11-74 (89) | BMI | 26.3 (25.9, 26.6) | 26.3 (25.7, 26.8) | 26.7 (25.1, 28.4) |  |  | 0.591 |  |
| n=652/232/26 | PG0 | 5.29 (5.25, 5.34) | 5.32 (5.25, 5.40) | 5.29 (5.07, 5.53) |  |  | 0.952 |  |
|  | 2hPG | 5.82 (5.69, 5.94) | 6.07 (5.85, 6.28) | 5.70 (5.12, 6.33) |  |  | 0.555 |  |
|  | INS0 | 41.1 (39.4, 42.8) | 42.3 (39.5, 45.3) | 37.9 (30.9, 46.6) |  |  | 0.413 |  |
|  | Ins inc | 29.9 (28.6, 31.3) | 29.9 (27.7, 32.2) | 26.6 (21.2, 33.3) |  |  | 0.310 |  |
| ABCC9 | BMI | 26.3 (26.0, 26.7) | 26.2 (25.7, 26.7) | 25.7 (24.4, 27.0) |  |  | 0.361 |  |
| IVS13-76 (100) | PG0 | 5.30 (5.25, 5.35) | 5.29 (5.22, 5.37) | 5.35 (5.17, 5.54) |  |  | 0.543 |  |
| n=586/246/41 | 2hPG | 5.87 (5.74, 6.00) | 5.93 (5.73, 6.14) | 5.60 (5.14, 6.09) |  |  | 0.255 |  |
|  | INS0 | 41.5 (39.7, 43.3) | 42.1 (39.4, 45.0) | 35.0 (29.7, 41.2) |  |  | **0.043** |  |
|  | Ins inc | 29.8 (28.4, 31.3) | 29.3 (27.2, 31.5) | 31.5 (26.3, 37.8) |  |  | 0.510 |  |
| LIPC | BMI | 26.1 (25.7, 26.4) | 26.8 (26.2, 27.3) | 26.3 (24.3, 28.3) | **0.029** | **0.046** |  |  |
| IVS1+49 (114)* | PG0 | 5.31 (5.26, 5.35) | 5.30 (5.22, 5.37) | 5.28 (5.00, 5.57) | 0.785 | 0.768 |  |  |
| n=609/259/17 | 2hPG | 5.87 (5.75, 6.00) | 5.86 (5.67, 6.06) | 6.16 (5.41, 7.02) | 0.941 | 0.793 |  |  |
|  | INS0 | 40.9 (39.1, 42.7) | 42.8 (40.1, 45.8) | 38.6 (29.9, 49.9) | 0.291 | 0.415 |  |  |
|  | Ins inc | 29.4 (28.0, 30.8) | 31.0 (28.8, 33.3) | 29.1 (22.0, 38.5) | 0.247 | 0.313 |  |  |
| PYY | BMI | 26.4 (26.1, 26.7) | 25.9 (25.2, 26.6) | 25.0 (21.6, 28.4) | 0.156 | 0.137 |  |  |
| IVS3+68 (123) | PG0 | 5.30 (5.26, 5.35) | 5.28 (5.19, 5.38) | 5.35 (4.89, 5.85) | 0.749 | 0.794 |  |  |
| n=736/147/6 | 2hPG | 5.87 (5.75, 5.98) | 5.90 (5.65, 6.17) | 5.09 (4.09, 6.33) | 0.978 | 0.813 |  |  |
|  | INS0 | 41.6 (40.0, 43.3) | 39.1 (35.8, 42.7) | 43.1 (28.1, 66.3) | 0.224 | 0.270 |  |  |
|  | Ins inc | 29.9 (28.7, 31.2) | 29.2 (26.5, 32.1) | 34.5 (21.5, 55.1) | 0.720 | 0.831 |  |  |
| INSR | BMI | 26.4 (26.0, 26.8) | 26.4 (25.8, 26.9) | 24.9 (23.3, 26.6) | 0.584 | 0.302 |  |  |
| IVS6+43 (131) | PG0 | 5.28 (5.23, 5.33) | 5.37 (5.29, 5.44) | 5.24 (5.02, 5.47) | 0.105 | 0.228 |  |  |
| n=586/245/27 | 2hPG | 5.83 (5.70, 5.97) | 6.08 (5.87, 6.30) | 5.78 (5.20, 6.42) | ***0.079*** | 0.166 |  |  |
|  | INS0 | 41.5 (39.7, 43.4) | 43.0 (40.1, 46.0) | 37.5 (30.5, 46.1) | 0.591 | 0.901 |  |  |
|  | Ins inc | 29.8 (28.4, 31.3) | 30.3 (28.1, 32.6) | 29.8 (23.8, 37.3) | 0.743 | 0.788 |  |  |
